# Higher Seed Rates Enlarge Effects of Wide-Belt Sowing on Canopy Radiation Capture, Distribution, and Use Efficiency in Winter Wheat

**DOI:** 10.3390/plants13070986

**Published:** 2024-03-29

**Authors:** Wen Li, Yulei Xiong, Jin Tong, Wen Lin, Jianfu Xue, Yuechao Wang, Zhiqiang Gao

**Affiliations:** College of Agriculture, Shanxi Agricultural University, Jinzhong 030801, China

**Keywords:** wide-belt sowing, seed rate, wheat yield, radiation interception, radiation use efficiency

## Abstract

The optimized winter wheat sowing method comprising wide-belt sowing (WBS) can improve the ears number and biomass to increase the grain yield, compared with conventional narrow-drill sowing (NDS). The seed rate and the interaction between the sowing method and seed rate also affect yield formation. However, the effects of the sowing method and seed rate, as well as their interaction on biomass production, particularly the interception of solar radiation (ISR) and radiation use efficiency (RUE), are unclear. A field experiment was conducted for two seasons in southern Shanxi province, China, using a split-plot design with sowing method as the main plot (WBS and NDS) and seed rate as the sub-plot (100–700 m^−2^). Our results showed that while WBS had a significant and positive effect, increasing the yield by 4.7–15.4%, the mechanism differed between seed rates. Yield increase by WBS was mainly attributed to the increase in total biomass resulting from both the promoted pre- and post-anthesis biomass production, except that only the increase in post-anthesis biomass mattered at the lowest seed rate (100 m^−2^). The higher biomass was attributed to the increased ISR before anthesis. After anthesis, the increased ISR contributed mainly to the increased biomass at low seed rates (100 and 200 m^−2^). In contrast, the increased RUE, resulting from the enhanced radiation distribution within canopy and LAI, contributed to the higher post-anthesis biomass at medium and high seed rates (400 to 700 m^−2^). The greatest increases in total biomass, pre-anthesis ISR, and post-anthesis RUE by WBS were all achieved at 500 seed m^−2^, thereby obtaining the highest yield. In summary, WBS enhanced grain yield by increasing ISR before anthesis and improving RUE after anthesis, and adopting relatively higher seed rates (400–500 m^−2^) was necessary for maximizing the positive effect of WBS, and thus the higher wheat yield.

## 1. Introduction

Wheat (*Triticum aestivum* L.) is one of the most important food crops throughout the world, with an annual harvested area of 221 million hectares and total yield of 771 million tons in 2021. World wheat yield in 2021 increased by 28.4%, compared to 2000 [1]. On one hand, the increased wheat yields were attributed to genetic improvements, such as enhanced photosynthesis, spike fertility, and the partitioning of available assimilates to the spikes [2,3]. On the other hand, intensified crop management practices involving improved fertilization, irrigation, control of pests and weeds, and different plant arrangements have critical roles in improving wheat yields [4,5,6]. In particular, the sowing method and seed rate can be changed to readily adjust the plant arrangement, and they have clear effects on crop growth and yield formation [7,8].

Many studies have shown that wide-belt precision planting enhances the yield, compared with conventional cultivation planting or narrow-drill sowing (NDS) [9,10,11]. Using low seed rates (less than 200 m^−2^), wide-belt precision planting changes the seed distribution from a narrow belt (3–5 cm) or a line into a wide belt (6–10 cm) for increasing the distance among single seed [12]. This high-yielding sowing technique required high land preparation quality and good soil fertility to ensure a high emergence rate and tillering capacity to produce sufficient ears for high yields. However, in many wheat producing regions in China, the lack of sufficient labor and large machinery, as well as inferior soil mechanical properties and fertility, limit the application and performance benefits of wide-precision planting. Thus, increasing the seed rate is applied to adapt wide-belt precision planting to compensate for the negative impacts of suboptimal land preparation and low soil fertility on the emergence rate and tillering capacity. For example, after this technique was introduced in Shanxi Province, China, the local farmers applied seed rates of 300–700 m^−2^ or even higher, and the yields increased by more than 10% [13]. Due to the higher seed rate, especially with suboptimal soil preparation, this sowing technique is referred to as wide-belt sowing (WBS). Previous studies have demonstrated that the yield increases under WBS are due to increases in the tillering capacity, ears number, and leaf area index (LAI), and thus biomass production [13,14,15].

The crop biomass is the product of intercepted solar radiation (ISR) and the radiation use efficiency (RUE) [16]. ISR is determined by the speed of canopy development and closure, leaf absorbance, canopy longevity, size, and architecture. Particularly, leaf area index (LAI) was usually recognized as a critical indicator to reflect radiation capture capacity [17,18]. RUE, namely utilization of solar radiation per unit of biomass, was the fundamental bottleneck to raising wheat yield [2] and was sensitive to the leaf N condition, especially specific leaf N content (SLN) [18,19,20]. Biomass increase can be achieved by improving either or both parameters. Zhao et al. [21] and Liu et al. [22] found that WBS could influence PAR interception by the canopy and the distribution of PAR within the canopy by affecting the morphological characteristics of the canopy, particularly the population size and leaf area. So far as we know, this is the case; however, little information is available about how different sowing methods affect RUE, as well as the associated physiological mechanisms.

Wheat crops are also highly sensitive to the seed rate [7,23,24,25]. Under relatively low seed rates, wheat plants produce stronger individual tillers or ears, but low nitrogen uptake and light interception by the canopy limits the capacity for biomass production, thereby restricting the yield performance. In addition, very high seed rates are not beneficial for increasing the uptake of nitrogen and intercepting more solar energy due to the limited nitrogen supply in the soil and already closed canopy, respectively. Thus, an optimum seed rate should be selected to maximize light interception and biomass production, and thus the grain yield.

Recently, Zheng et al. found a significant interaction between the effects of the sowing method and seed rate on the grain yield in a trial conducted over two seasons [6]. The positive effect of WBS depended significantly on the seed rate. The seed rate at which the top yield was obtained under WBS was higher than NDS. Similarly, the preliminary observations in our field experiments and in farmers’ fields in southern Shanxi province also suggested a strong interaction between the effects of the sowing method and seed rate on the grain yield. Zheng et al. systematically investigated the underlying mechanism in terms of the yield components, and N uptake and utilization, but lacked insights into biomass production, ISR and RUE, which determined the crop yield potential and drove actual yield performance [17,26].

In the present study, the yield and yield components, population size and individual productivity, biomass production and partitioning, canopy solar radiation interception and distribution, RUE, LAI, and SLN were determined for winter wheat in a field experiment conducted for two growing seasons under two sowing methods (NDS and WBS) and seven seed rates (100 to 700 m^−2^, with an interval of 100 m^−2^). The objectives of this study were: (1) to determine how the sowing method, seed rate, and their interaction affected the yield, biomass accumulation, and capture and use of solar radiation; and (2) to identify the associated agronomic and physiological mechanisms that were involved in the yield increases by WBS at various seed rates. In addition to hypothesizing that WBS can enhance the solar radiation interception and/or RUE, and thereby promote biomass production and grain yield, we also hypothesize that the importance of solar radiation interception and RUE differ at various seed rates.

## 2. Results

### 2.1. Yield and Yield Components

**Figure 1:**
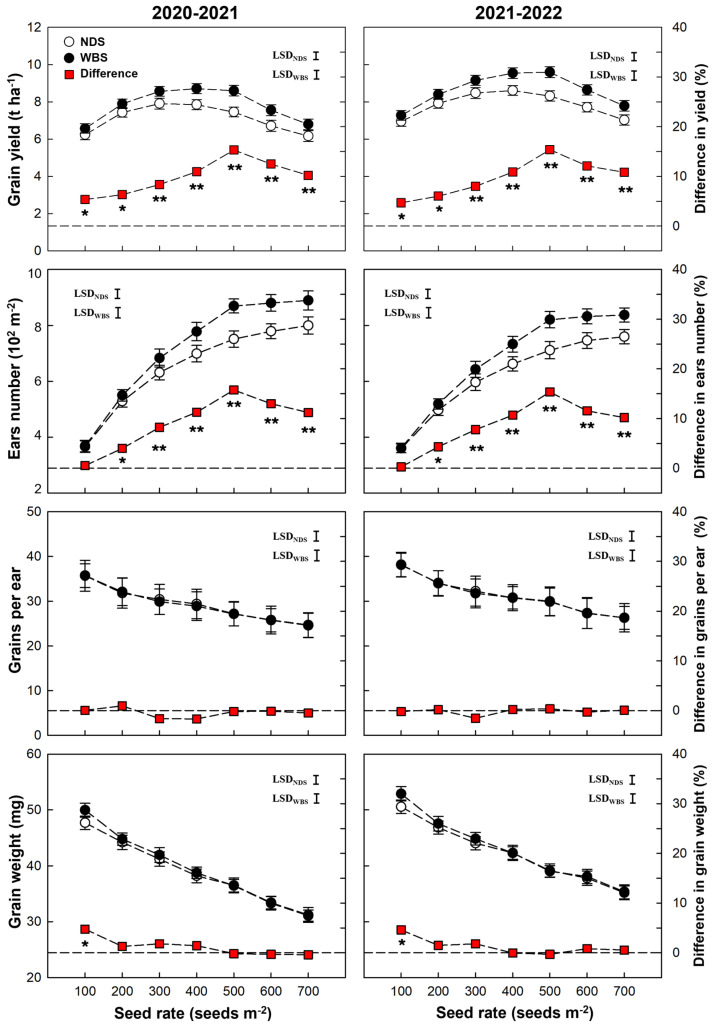
Grain yield and yield components of winter wheat with narrow drill sowing (NDS) and wide-belt sowing (WBS) in the 2020–2021 and 2021–2022 growing seasons. Data are means and error bars are SE (n = 4). The dashed reference line indicates that the difference percentage in yield or yield component between WBS and NDS is zero. The * and ** indicate there is significant difference between WBS and NDS at the same seed rate, according to Student’s *t*-test at *α* = 0.05 and 0.01, respectively.

The grain yield was significantly affected by the sowing method and seed rate (Table 1). In the 2020–2021 season, the highest yields of 7.91 and 8.71 t ha^−1^ were obtained at SR300 and SR400 under NDS and WBS, respectively (Figure 1). In the 2021–2022 season, SR400 and SR500 produced the greatest yields of 8.61 and 9.60 t ha^−1^ under NDS and WBS, respectively. The grain yield increased initially and then decreased with the increasing seed rate under both sowing methods. The clear yield decrease was observed when the seed rate increased from 400 to 500 m^−2^, whereas it occurred as the seed rate increased from 500 to 600 m^−2^ under WBS. Thus, the effect of the interaction between the sowing method and seed rate on the grain yield was significant. Furthermore, the difference in yield between sowing methods increased from 4.7–5.4% at SR100, peaked to 15.3–15.4% at 500 seeds m^−2^, and then decreased to 10.2–10.8% at SR700 in the two seasons.

All yield components (ears per m^2^, grains per ear, and grain weight) were significantly affected by the seed rate in both seasons (Table 1). However, only the ears per m^2^ was also affected by the sowing method and the interaction between the sowing method and seed rate. The ears per m^2^ tended to increase as the seed rate increased under both sowing methods in both seasons. WBS significantly increased the ears per m^2^ at all seed rates, except for SR100. The highest difference in the ears number was obtained at SR500 in both seasons. There was no significant difference in the grains per ear and grain weight between WBS and NDS at any seed rate, except the grain weight was 4.6–4.7% higher (*p* < 0.05) under WBS at SR100.

### 2.2. Population Size and Individual Productivity of Stems

**Figure 2:**
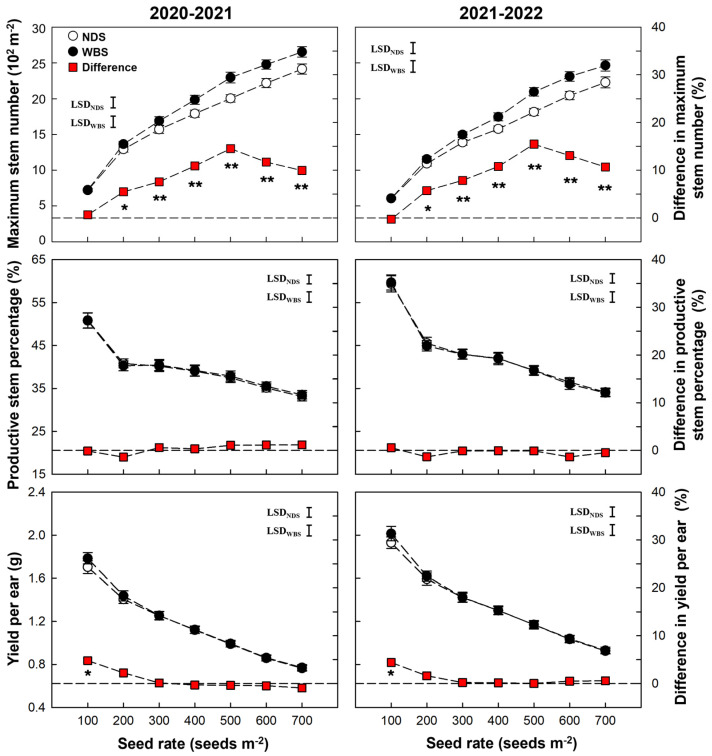
Maximum number of stems, percentage of effective stems and yield per effective stem in two growing seasons. Data are means and error bars are SE (n = 4). The dashed reference line indicates that the difference percentage in a specific trait between WBS and NDS is zero. The * and ** indicate there is significant difference between wide-belt sowing (WBS) and narrow-drill sowing (NDS) at the same seed rate, according to Student’s *t*-test at *α* = 0.05 and 0.01, respectively.

The increase in the ears per m^2^ under WBS was only attributed to the increased maximum stem number (Figure 2). When the seed rate exceeded 200 m^−2^, the maximum stem numbers were 7.6–14.5% and 6.4–15.5% higher under WBS than NDS during the 2020–2021 and 2021–2022 seasons, respectively. There was no significant difference in the productive stem percentage and yield per productive stem between WBS and NDS at any seed rate, except the yield per productive stem was 4.4–4.8% (*p* < 0.05) higher under WBS at SR100.

### 2.3. Biomass Production and HI

**Figure 3:**
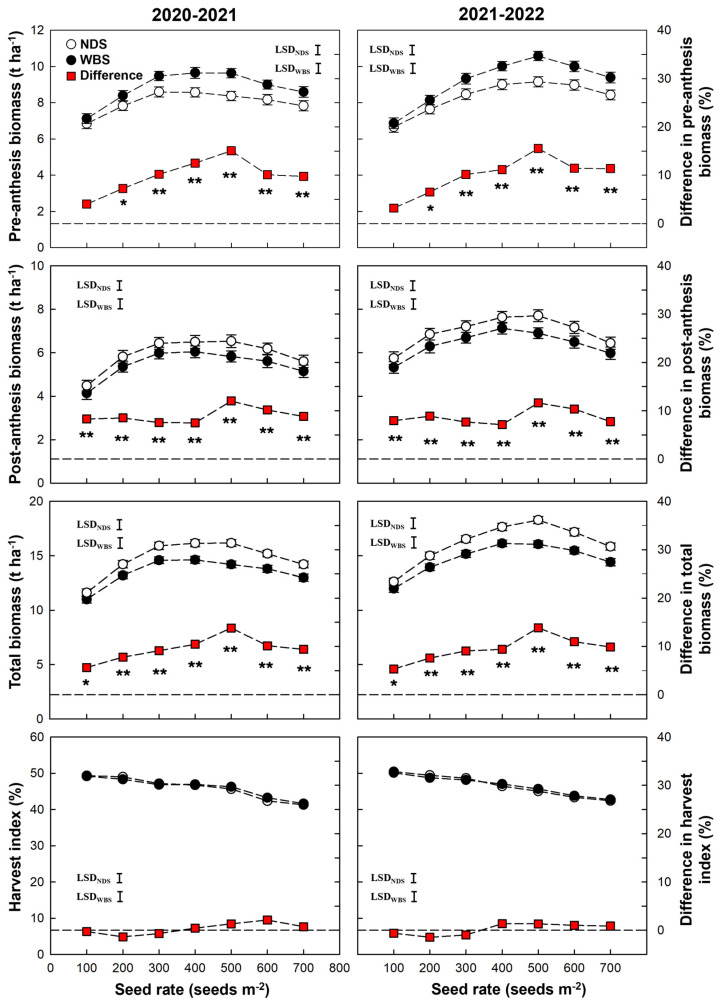
Biomass production and harvest index of winter wheat with narrow-drill sowing (NDS) and wide-belt sowing (WBS) in two growing seasons. Data are means and error bars are SE (n = 4). The dashed reference line indicates that the difference percentage in biomass or harvest index between WBS and NDS is zero. The * and ** indicate there is significant difference between WBS and NDS at the same seed rate, according to Student’s *t*-test at *α* = 0.05 and 0.01, respectively.

All of Biomass_pre_, Biomass_post_, and Biomass_total_ increased initially and then decreased as the seed rate increased in both seasons (Figure 3). At all seed rates, Biomass_pre_, Biomass_post_, and Biomass_total_ were 6.5–15.5%, 7.1–12.0%, and 5.3–13.9% higher under WBS, compared with NDS, respectively, except for Biomass_pre_ at SR100. The highest increase in biomass production was obtained at SR500 in both seasons. HI tended to decrease as the seed rate increased under both sowing methods and no difference was observed between WBS and NDS at any seed rate in either season.

### 2.4. Radiation Interception and RUE

**Figure 4:**
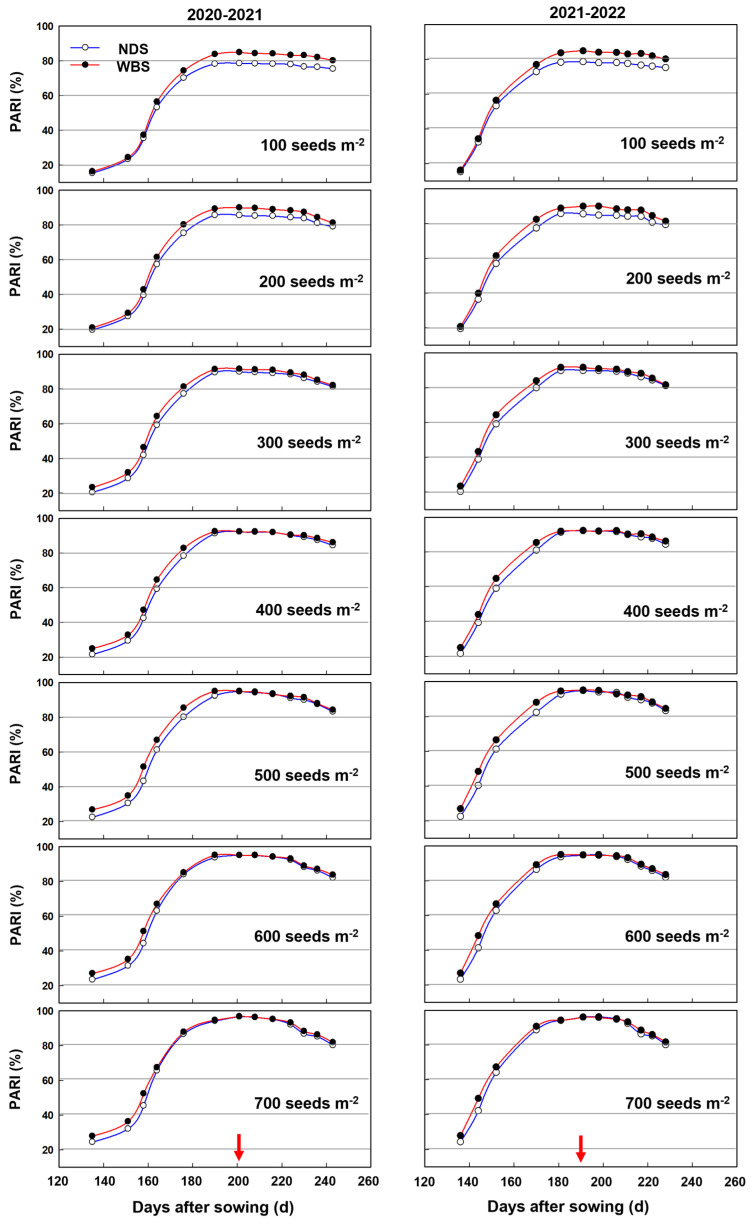
Canopy PAR interception ratio (PARI) dynamic after winter dormancy in the 2020–2021 and 2021–2022 growing seasons. The downward arrow indicates the date of anthesis.

At seed rates of 100–300 m^−2^, the post-anthesis PARI under WBS was higher than NDS, and when the seed rate was higher, there was a smaller difference (Figure 4). In addition, the obvious difference in PARI between sowing methods appeared at 163, 158, and 133 d after sowing under seed rates of 100, 200, and 300 m^−2^, respectively. At seed rates of 400–700 m^−2^, there was no obvious difference in post-anthesis PARI between sowing methods, and the difference in PARI disappeared earlier when the seed rate increased from 400 to 700 m^−2^.

**Figure 5:**
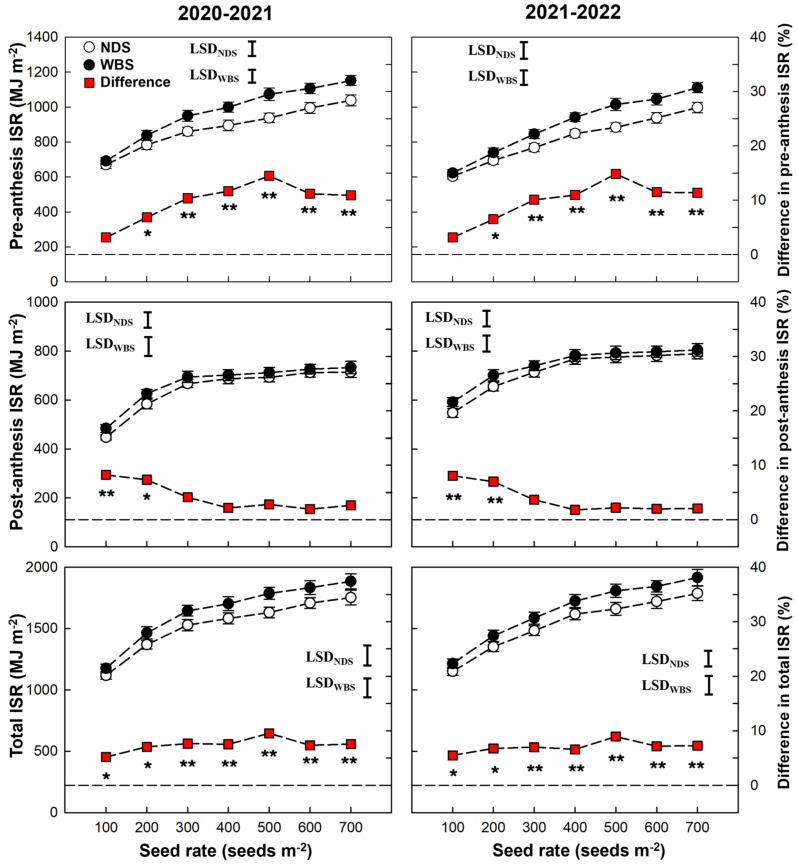
Intercepted solar radiation (ISR) of winter wheat with narrow-drill sowing (NDS) and wide-belt sowing (WBS) in two growing seasons. Data are means and error bars are SE (n = 4). The dashed reference line indicates that the difference percentage in ISR between WBS and NDS is zero. The * and ** indicate there is significant difference between WBS and NDS at the same seed rate, according to Student’s *t*-test at *α* = 0.05 and 0.01, respectively.

In general, as the seed rate increased, the pre-anthesis and total ISR tended to continuously increase, but the post-anthesis ISR increased when the seed rate increased from 100 to 300 and from 100 to 400 m^−2^ in 2020–2021 and 2021–2022 seasons, respectively, and then stabilized (Figure 5). There was no significant difference between WBS and NDS at SR100 in either season. For pre-anthesis ISR, significant increases by WBS were 6.8–14.5% and 6.5–14.8% in the 2020–2021 and 2021–2022 seasons, respectively, and the highest increase occurred at SR500. For post-anthesis ISR, significant increases by WBS, of 8.1–8.2% and 7.0–7.3%, were observed at SR100 and SR200, respectively. For the total ISR, significant increases by WBS were recorded for any rare seed, with the highest percentage of 8.9–9.5% at SR500.

**Figure 6:**
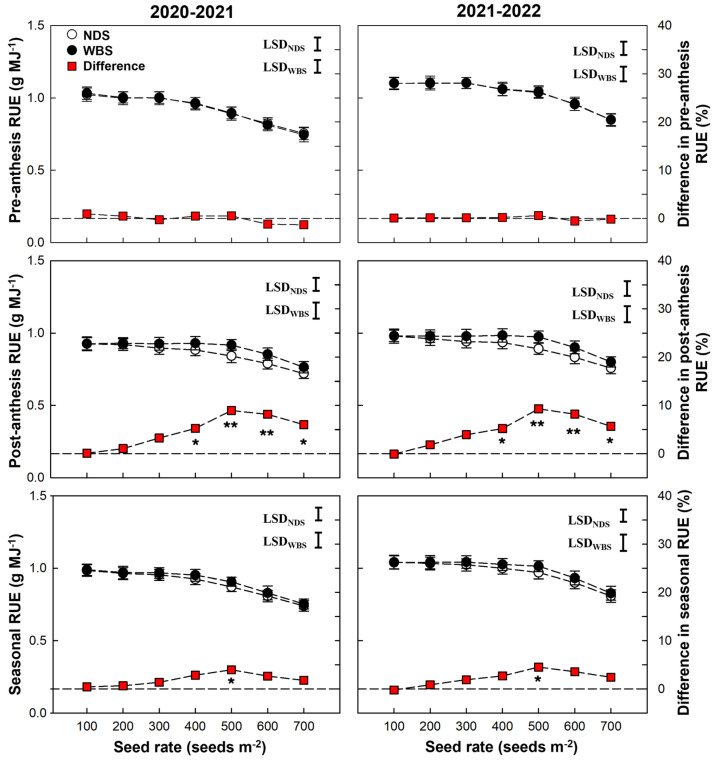
Radiation use efficiency (RUE) of winter wheat with narrow-drill sowing (NDS) and wide-belt sowing (WBS) in two growing seasons. Data are means and error bars are SE (n = 4). The dashed reference line indicates that the difference percentage in RUE between WBS and NDS is zero. The * and ** indicate there is significant difference between WBS and NDS at the same seed rate, according to Student’s *t*-test at *α* = 0.05 and 0.01, respectively.

In both seasons, the RUE during pre-anthesis, post-anthesis, and the entire season tended to decrease when the seed rate increased from 100 to 700 m^−2^ (Figure 6). There was no difference in pre-anthesis RUE between WBS and NDS at any seed rate. However, significant increases of 5.2–9.0% and 5.3–9.2% in post-anthesis RUE by WBS were obtained in the 2020–2021 and 2021–2022 seasons, respectively. The seasonal RUE increased significantly under WBS, compared to NDS, only at SR500 in each season.

### 2.5. PAR Distribution within Wheat Canopy at Anthesis

**Figure 7:**
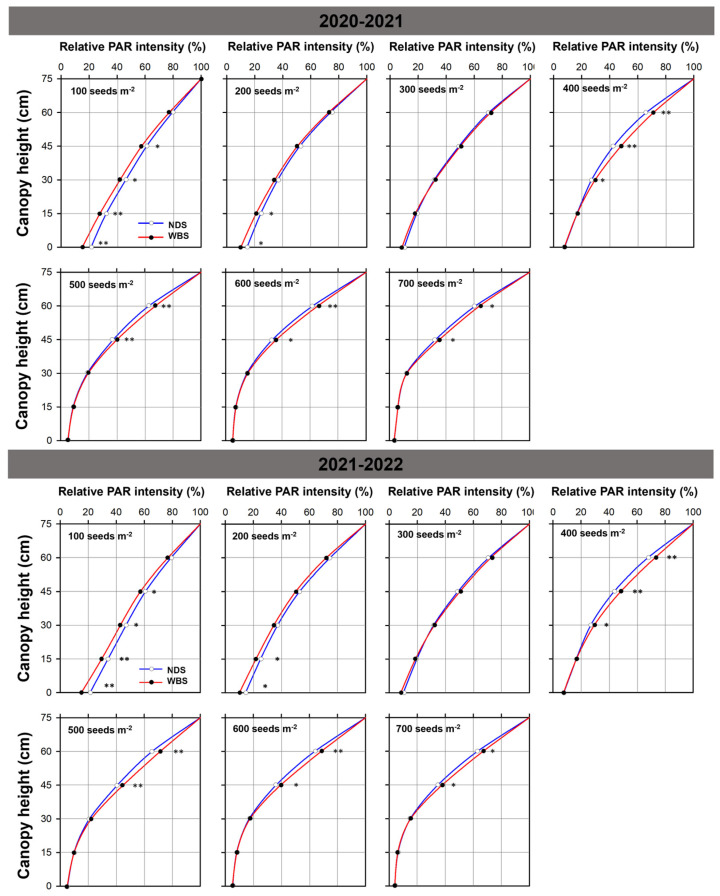
Relative photosynthetically active radiation (PAR) intensity at different height within the canopy in the 2020–2021 and 2021–2022 growing seasons. Relative PAR intensity presents the percentage of the intensity of PAR at a specific height within the canopy to the intensity above the canopy. The * and ** indicate there is significant difference between narrow-drill sowing (NDS) and wide-belt sowing (WBS) at the same seed rate, according to Student’s *t*-test at *α* = 0.05 and 0.01, respectively.

At SR100 and SR200, the relative PAR intensity at the soil surface, or PAR transmission ratio, decreased by 29.2–30.4% under WBS, compared to NDS at anthesis (Figure 7). At SR300, the relative PAR intensity did not differ significantly between methods at any canopy height. At the seed rates of 400 to 700 m^−2^, the relative PAR intensity increased at canopy heights of 60 and 45 cm. At the canopy height of 60 cm, the relative PAR intensities were 5.0–7.7% and 6.4–8.9% higher under WBS than NDS in the 2020–2021 and 2021–2022 seasons, respectively, and the corresponding increases at the height of 45 cm were 7.0–12.4% and 9.1–10.8%. In addition, at the height of 30 cm, the relative PAR intensities were higher (by 9.5–11.6%, *p* < 0.05) under WBS than NDS, at 400 seeds m^−2^ in both seasons.

### 2.6. Leaf Area Index and Specific Leaf Nitrogen at Anthesis

**Figure 8:**
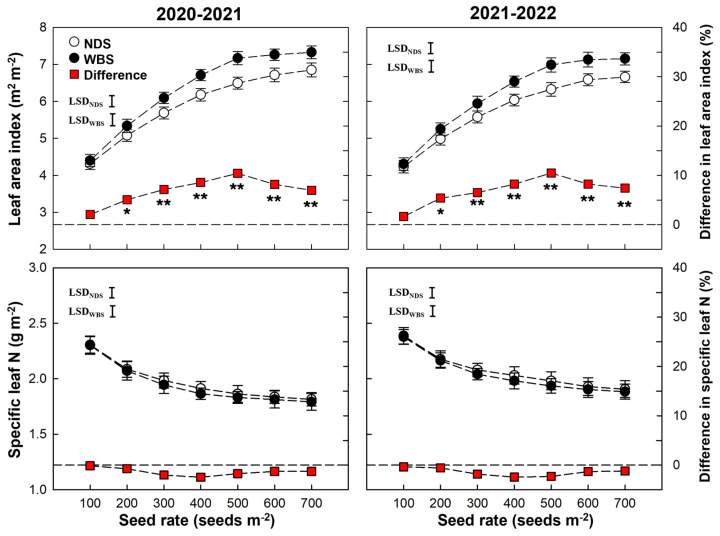
Leaf area index and specific leaf N at anthesis of winter wheat with narrow-drill sowing (NDS) and wide-belt sowing (WBS) in two growing seasons. Data are means and error bars are SE (n = 4). The dashed reference line indicates that the difference percentage in leaf area index or specific leaf N between WBS and NDS is zero. The * and ** indicate there is significant difference between WBS and NDS at the same seed rate, according to Student’s *t*-test at *α* = 0.05 and 0.01, respectively.

The LAI at anthesis was significantly affected by the sowing method, seed rate, and their interaction, but the SLN at anthesis was only significantly affected by the seed rate (Table 1). Under both sowing methods, LAI increased as the seed rate increased and then statured at SR500, whereas SLN tended to decrease in both seasons (Figure 8). At SR100, there was no significant difference in LAI between WBS and NDS in either season. However, when the seed rate exceeded 100 m^−2^, the LAI values were 5.1–10.4% and 5.4–10.5% higher under WBS than NDS in the 2020–2021 and 2021–2022 seasons, respectively. By contrast, the SLN values were slightly lower, but not significantly (0.1–2.5%, *p* > 0.05), under WBS in both seasons.

### 2.7. Correlation Analysis

**Figure 9:**
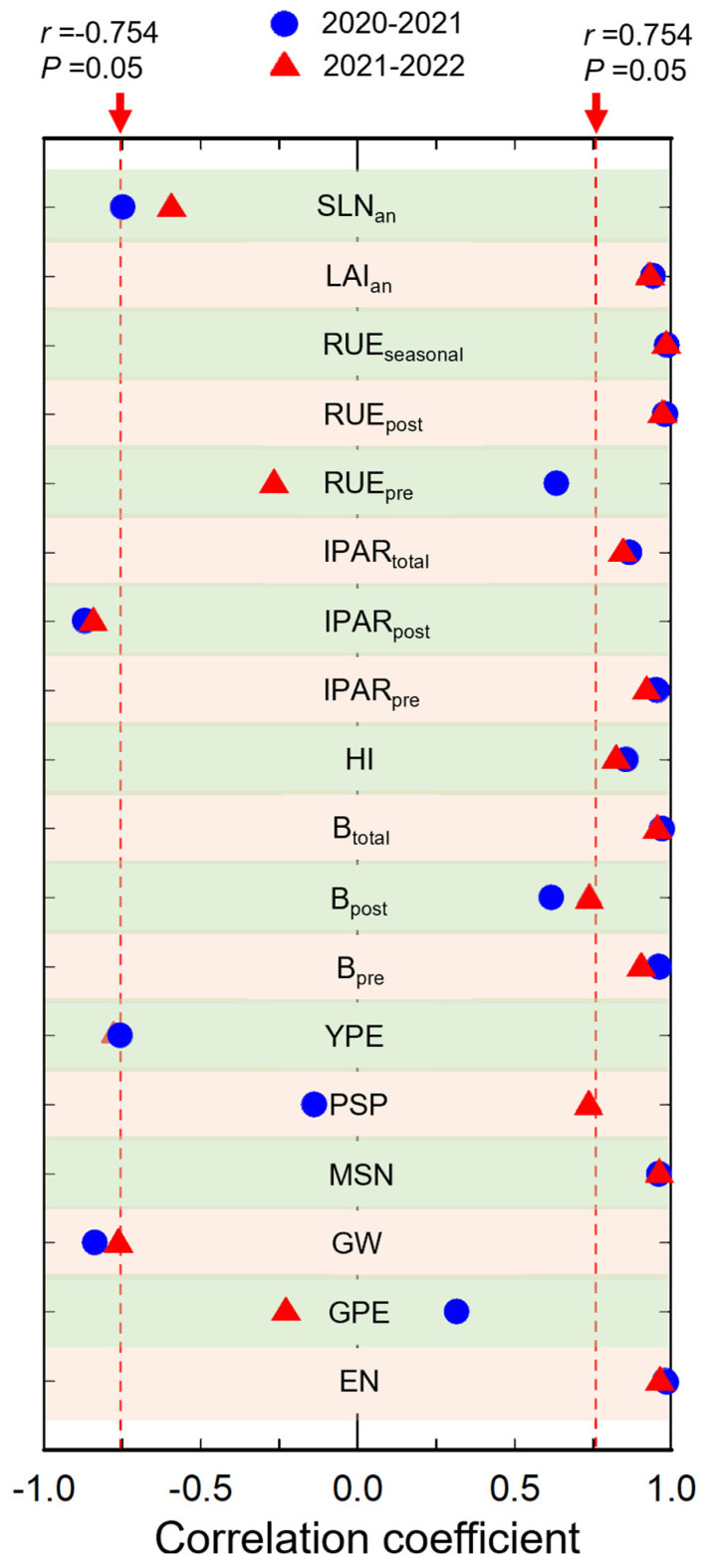
The correlation coefficient (*r*) of difference in yield and difference in yield-related traits in two growing seasons, based on Pearson correlation analysis. The dashed reference line indicates the critical *r* value at *p* = 0.05. EN, ears number; GPE, grains per ear; GW, grain weight; MSN, the maximum stem number; PSP, productive stem percentage; YPE, yield per ear; B_pre_, pre-anthesis biomass; B_post_, post anthesis biomass; B_total_, total biomass; HI, harvest index; ISR_pre_, pre-anthesis intercepted solar radiation; ISR_post_, post-anthesis intercepted solar radiation; ISR_total_, total intercepted solar radiation; RUE_pre_, pre-anthesis radiation use efficiency; RUE_post_, post-anthesis radiation use efficiency; RUE_seasonal_, seasonal radiation use efficiency; LAI_an_, leaf area index at anthesis; SLN_an_, specific leaf nitrogen content at anthesis.

In both seasons, the difference in the grain yield between WBS and NDS was significantly positively correlated with the differences in the ears number, the maximum stems number, pre-anthesis, post-anthesis and total biomass, pre-anthesis and total ISR, post-anthesis and seasonal RUE, and LAI at anthesis; but was significantly and negatively correlated with the difference in grain weight (Figure 9).

## 3. Discussion

It has been widely recognized that the more uniform crop spatial patterns can boost crop yield, with the reduced row distance and the increased within-row uniformity playing vital roles in this regard [27]. However, the effects on yield usually varied depending on crop sowing density (i.e., seed rate) [28,29,30]. The optimized winter wheat sowing method comprising WBS combining the two above aspects significantly enhanced the yield, but the yields and the underlying agronomic and physiological mechanisms differed between seed rates. Our results showed that the increase in the yield under WBS, compared with NDS, was greater at a higher seed rate. However, when the seed rate reached 600 and 700 m^−2^, the increase in the yield was decreased. High yields were obtained at SR300 and SR400 under NDS, but the high yields under WBS were obtained at relatively greater seed rates of 400–500 m^−2^, and similar results were reported by Zheng et al. (2020) [6]. These results suggest that relatively higher seed rates are suitable for enhancing the positive effect of WBS to maximize yields.

It is widely accepted that the increase in the yield under WBS, compared with NDS, is mainly due to the increased ears number [13,21,31,32]. In the present study, the improvement in ears number increased initially and then decreased as the seed rate increased under WBS, and this trend was similar to that in the yield (Figure 3). Furthermore, the correlation analysis showed at least 93.9% of the variation in the increase in yield under WBS could be explained by the increase in ears number (*p* < 0.01, Figure 9). The ears number is determined by the tillering capacity (maximum stem number) and the productive stem percentage [33]. Previous studies also showed that WBS reduced intraspecific competition for resources and growing space, thereby resulting in the production of more stems [22,34]. In the present study, wheat plants produced 5.5–15.5% more stems under WBS, compared with NDS, at seed rates of 200–700 m^−2^ but without the cost of depressed productive stem percentage. On the other hand, it should be noted that wheat plants produced more ears under WBS but without a decrease in the yield per ear. In particular, at a low seed rate of 100 m^−2^, the yield only increased by 4.7–5.4% under WBS, which was solely attributed to the increase in the grain weight or yield per ear instead of the ears number. A previous trial conducted by Liu et al., using a density of 150 plants m^−2^, also found a relatively low yield increase of 2.7–4.6% under WBS, compared with NDS [22]. The omission of intraspecific competition among individuals at a seed rate of 100 m^−2^ probably explains the insignificant improvement in the population size (maximum stem number and ears number) by optimizing the seed distribution. These results suggest that optimizing the seed distribution in the field allowed the establishment of a greater wheat population size, while also maintaining a considerable individual productivity at seed rates of 200 m^−2^ and higher.

The crop yield is determined by biomass production and HI, but greater yields are obtained by increasing the net primary productivity of modern rice, maize, and wheat varieties [3,35]. In our previous study, we showed that the biomass, rather than HI, explained the increased grain yield under WBS at 300 plants m^−2^ [13], as recognized by Chen et al. [36,37]. In the present study, our results confirmed the positive effect of WBS on biomass production, and correlation analysis showed that the increase in total biomass under WBS could explain at least 91.3% of the variation in the increase in yield (*p* < 0.01, Figure 9). The increased total biomass under WBS at SR100 was mainly attributed to the increased post-anthesis biomass, whereas the biomass production during the pre- and post-anthesis periods were both important at seed rates of 200–700 m^−2^. The crop biomass production is the product of ISR and RUE, which indicates the capacities of the canopy in capturing and using solar radiation to produce biomass, respectively [19,38,39]. At all seed rates, the significant increases in the total biomass under WBS were mainly attributed to the significant increases in total ISR, as well as the significant increase in the seasonal RUE at 500 m^−2^. Both the highest increase in the total ISR and unique significant increase in the seasonal RUE contributed to the greatest improvement in the total biomass under WBS at SR500.

The importance of ISR or RUE for the increases in biomass during the pre- and post-anthesis periods differed between seed rates. In the pre-anthesis period, the significant increases in the biomass at 200–700 seeds m^−2^ were mainly due to the significant increase in ISR, whereas the lack of any significant effect of WBS on ISR or RUE led to no significant difference in the biomass at SR100. In the post-anthesis period, the increased ISR made the main contribution to the increase in the biomass at SR100 and SR200. Relatively small increases (3.3–4.1%, *p* > 0.05) in ISR and RUE contributed to the significant increase in the biomass at SR300. At seed rates of 400–700 m^−2^, the increases in the biomass were mainly due to the increase in RUE. The increased population size and higher LAI could explain the enhanced pre-anthesis ISR. Olsen and Weiner stated that the uniform planting pattern could enhance LAI by 33–37% [40].

Previous studies also showed that seed arrangement could regulate the capture and distribution of PAR within the canopy [41,42,43]. At relatively low densities of 222 and 150 plants m^−2^, WBS narrowed the space between seedling belts and extended the belts to produce a more homogeneous canopy, and thus a higher proportion of PAR was intercepted at anthesis, compared with NDS [21,22]. In the present study, we attributed the more homogeneous canopy under WBS mainly to the significantly increased PAR interception ratio after anthesis at SR100, because no greater population size and LAI was observed. At SR200, besides the more homogeneous canopy, the enlarged population size and LAI under WBS also helped improved the PAR interception ratio after anthesis. At SR300 and higher, the increases in the population size and LAI under WBS improved the pre-anthesis ISR by 10.1–14.8%, whereas there was only a small and not significant improvement (1.8–4.1%) in the post-anthesis ISR due to the almost closed canopy since anthesis, even under NDS. These results indicated that, at extremely low seed rate, WBS mainly improved the post-anthesis ISR, which was due to the more homogeneous canopy; whereas the enlarged population size or LAI were more important at medium or high seed rates during the pre-anthesis period.

It is widely acknowledged that SLN is a critical physiological trait that affects the potential capacity for leaf photosynthesis, and thus the canopy RUE [44,45]. However, our results showed that WBS significantly increased the post-anthesis RUE, despite the lack of a significant increase in the canopy SLN at anthesis at seed rates of 400–700 m^−2^. These results suggested that the SLN, or the potential capacity for leaf photosynthesis, was not responsible for the increased post-anthesis RUE under WBS. Yan et al. [46] and Chen et al. [47] showed that an enhanced PAR distribution within the canopy could be beneficial for increasing the RUE and wheat yield. Our results showed that WBS significantly increased the PAR intensity in the sub-top and middle canopy layers. The increased PAR intensity would enhance the actual photosynthesis rate in the sub-top and middle canopy layers, where leaves usually received insufficient light intensity to fully perform their photosynthetic capacity [48]. In addition to the enhanced actual photosynthetic rate, the greater LAI (photosynthetic area) also contributed to the increase in post-anthesis RUE under WBS. Future studies should focus on understanding why WBS allowed more PAR to be transmitted into the deeper layers despite the larger total leaf area—here it is possible that angle of inclination and size of the upper leaves may have important roles.

## 4. Materials and Methods

### 4.1. Site Description

Field experiments were conducted at Gucheng Village (35°43′ N, 111°44′ E), Tangxing Town, Yicheng County, Shanxi Province, in two winter wheat growing seasons during 2020–2021 and 2021–2022. The site has a typical semi-arid, warm temperate, and continental monsoon climate (Köppen classification), where the dominant cropping system is winter wheat and summer corn rotation. Experiments were conducted in the same field in the two growing seasons, where the previous crop was summer corn with normal and uniform management (similar with local farmers). Five replicate soil samples were randomly collected from the 0–20 cm and 20–40 cm soil layers for soil analysis before applying the basal fertilizer in 2020 and 2021. The soil type was classified as silty clay loam according to the USDA Soil Taxonomy, with a pH of 8.33–8.41, organic matter content of 19.52–20.38 g kg^−1^, total N content of 0.94–1.01 g kg^−1^, alkaline N content of 48.25–50.07 mg kg^−1^, Olsen P content of 19.23–19.55 mg kg^−1^, and available K content of 171.87–174.24 mg kg^−1^ in 0–20 cm soil. For 20–40 cm soil, the pH valued 8.36–8.46, organic matter content was 16.14–16.67 g kg^−1^, total N content was 0.81–0.92 g kg^−1^, alkaline N content was 37.36–40.11 mg kg^−1^, Olsen P content was 17.41–18.37 mg kg^−1^, and available K content was 161.44–169.17 mg kg^−1^.

**Figure 10:**
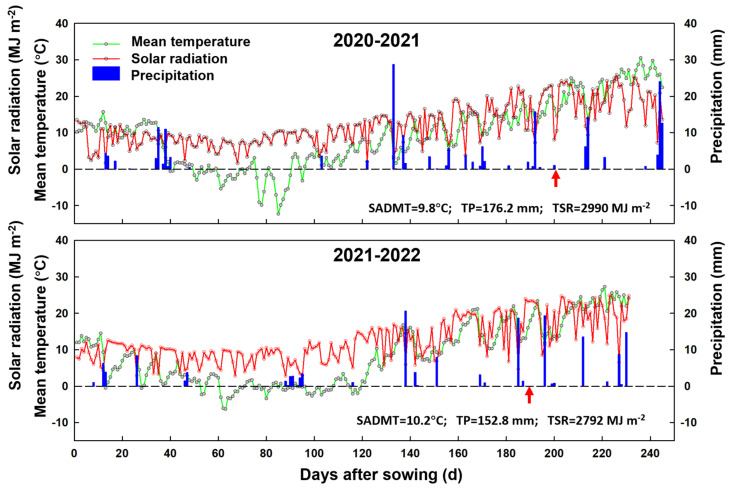
Daily mean temperature, solar radiation and precipitation recorded from sowing to maturity in the 2020–2021 and 2021–2022 growing seasons. The upward arrow indicates the date of anthesis; SADMT, seasonal average daily mean temperature; TP, total precipitation; TSR, total solar radiation.

Climate parameters, comprising the daily minimum temperature, maximum temperature, incident solar radiation, and precipitation during the growing period from sowing to maturity in both seasons, were collected from a weather station (AWS 800, Campbell Scientific, Inc., Logan, UT, USA) located about 50 m from the experimental field. The developmental stages of wheat plants were recorded using the Zadoks scale [49]. The seasonal average daily mean temperature, total precipitation, and incident solar radiation in the growing seasons during 2020–2021 and 2021–2022 were 9.8 °C and 10.3 °C, 176.2 mm and 132.3 mm, and 2990 MJ m^−2^ and 2792 MJ m^−2^, respectively (Figure 10).

### 4.2. Experimental Design and Crop Management

**Figure 11:**
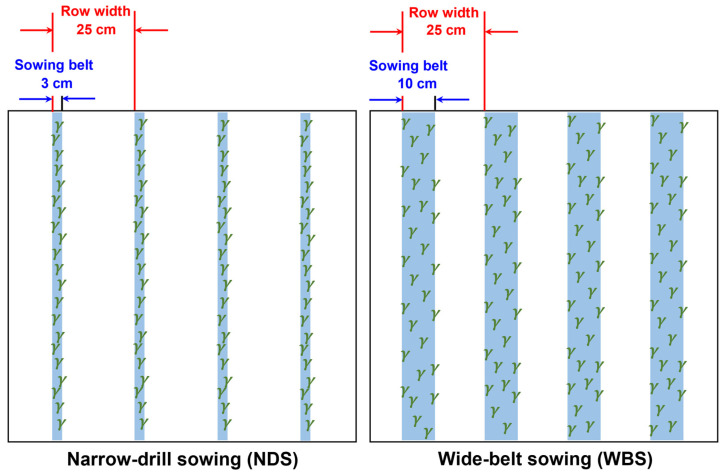
The sketch maps of narrow-drill sowing (NDS) and wide-belt sowing (WBS) used in this study.

The treatments were laid out in a split-plot design with four replicates. Sowing method was designated as the main plot and seed rate as the sub-plot. Each subplot measured 6.0 m in length and 2.0 m in width. The sowing methods (Figure 11) comprised conventional NDS (sowing belt and row widths of 3 cm and 25 cm, respectively) and WBS (sowing belt and row widths of 10 cm and 25 cm, respectively), which is used widely for wheat production in the study region. The widely planted wheat cultivar Zhongmai175 was selected. For both sowing methods, the seven seed rates were 100, 200, 300, 400, 500, 600, and 700 m^−2^ (SR100, SR200, SR300, SR400, SR500, SR600, and SR700). Although sowing is always done with a planter in farmers’ fields in the Shanxi province and on a national scale, a preliminary study showed that the designed sowing densities cannot be obtained accurately with planters, so the sowing was done manually in this study. The plant density was counted at the three-leaf stage (Zadoks code 13) and the ratios of plants relative to seeds were 93.7% and 92.0% in 2020 and 2021, respectively, and there was no significant difference between the treatments in each year.

Wheat crops were sowed on 15 October and 24 October in 2020 and 2021, respectively. In 2021, the heavy rain in early October made the soil quite wet and not suitable for sowing, and thus sowing was delayed for 9 days, compared with 2020.

N fertilizer was applied as urea (46% N), where 60% (135 kg N ha^−1^) of the N fertilizer was applied before sowing, with 40% (90 kg N ha^−1^) as topdressing fertilizer during the jointing stage (Zadoks code 32). Before sowing, phosphate, in the form of calcium super-phosphate (16% P_2_O_5_), was applied at 150 kg P_2_O_5_ ha^−1^ and potassium, in the form of potassium chloride (52% K_2_O), was applied at 90 kg K_2_O ha^−1^. Each plot was irrigated three times in the first season and two times in the second season. Irrigation water was applied (60 mm, 0.72 m^3^ plot^−1^) before winter dormancy (Zadoks code 23) in the first growing season and at the jointing and anthesis (Zadoks code 65) stages in both growing seasons. Irrigation water was supplied by a movable sprinkler system and the amount of water was measured using a flow meter. The field was kept free of diseases and pests. Weeds were controlled by spraying with herbicide three times in each experimental season.

### 4.3. Sampling and Measurements

The maximum number of stems (sum of main stems and tillers) was counted in four typical and central rows over a length of 1 m (1.00 m^2^) at jointing. The productive stems percentage (PSP) was calculated as the ratio of the ears number at maturity (Zadoks code 91), relative to the maximum number of stems at jointing. At anthesis, wheat plants were sampled from a typical and central row over a length of 0.5 m (0.125 m^2^). The green leaves were separated and measured using a leaf area meter (LI-3100C, LI-COR, Lincoln, NE, USA), before calculating the LAI as follows.

(1) $$LAI\left(m^{2}m^{-2}\right)=\frac{\mathrm{Leaf}\;\mathrm{area}}{\mathrm{Sampled}\;\mathrm{land}\;\mathrm{area}}$$

After measuring the leaf area, the leaves were oven dried to constant weight at 80 °C and then ground for analysis. The leaf N concentration (N mass per unit dry weight, mg g^−1^) was determined using an elemental analyzer (Rapid N Exceed, Elementar, Langenselbold, Germany). The specific leaf N (SLN, g m^−2^) was calculated as follows.

(2) $$SLN\left(gm^{-2}\right)=\frac{\mathrm{Leaf}\;N\;\mathrm{concentration}\times \mathrm{Leaf}\;\mathrm{mass}}{\mathrm{Sampled}\;\mathrm{leaf}\;\mathrm{area}}$$

At anthesis, the dry weights of whole plants were measured after oven drying to constant weight at 80 °C, to determine the pre-anthesis biomass (Biomass_pre_). At maturity, plants sampled from a typical and central row over a length of 0.5 m (0.125 m^2^) were manually divided into grain and straw. Dry weights were measured for the grain and straw after oven drying to constant weight at 80 °C. The total biomass at maturity (Biomass_total_) was the total dry weight of grain and straw. The post-anthesis biomass (Biomass_post_) was calculated as follows.

(3) $${\mathrm{Biomass}}_{\mathrm{post}}\left(kg{ha}^{-1}\right)={\mathrm{Biomass}}_{\mathrm{total}}-{\mathrm{Biomass}}_{\mathrm{pre}}$$

The harvest index (HI) was calculated as follows.

(4) $$\mathrm{HI}\left(\%\right)=\frac{\mathrm{Yield}\times 0.87}{{\mathrm{Biomass}}_{\mathrm{total}}}\times 100$$

At maturity, wheat ears were cut from an area of 1.0 m^2^ (length of 1.00 m in four typical rows) in the center of each plot, and the number of productive ears that produced at least five grains was recorded. The grain yield was adjusted to a standard moisture content of 0.130 g H_2_O g^−1^ fresh weight. The grain moisture content was measured using a digital moisture tester (PM8188A, Kett Electric Laboratory, Tokyo, Japan). Three sub-samples weighing 50.00 g were taken from the grain samples to count the number of grains and calculate the grain weight, which was also adjusted to a moisture content of 13.0%. The number of grains per ear was calculated as follows.

(5) $$\mathrm{Grains}\;\mathrm{per}\;\mathrm{ear}=\frac{\mathrm{Grains}\;\mathrm{yield}}{\mathrm{Grains}\;\mathrm{weight}\times \mathrm{Ears}\;\mathrm{number}}$$

Canopy PAR interception was measured during the growing seasons. The measurements were performed between 1100 h and 1300 h at an interval of 10–15 days using a linear PAR ceptometer (AccuPAR LP-80, Decagon Devices Inc., Pullman, WA, USA). In each plot, the transmitted PAR intensity was measured by placing the light bar vertical to rows and slightly above the soil surface. The PAR intensity above the canopy was recorded immediately after measuring the transmitted PAR intensity. Six pairs of PAR intensity measurements were recorded below and above the canopy. The canopy PAR interception ratio (PARI) was calculated as follows.

(6) $$\mathrm{PARI}\left(\%\right)=\frac{\mathrm{PAR}\;\mathrm{intensity}\;\mathrm{above}\;\mathrm{canopy}-\mathrm{PAR}\;\mathrm{intensity}\;\mathrm{slightly}\;\mathrm{above}\;\mathrm{the}\;\mathrm{soil}\;\mathrm{surface}}{\mathrm{PAR}\;\mathrm{intensity}\;\mathrm{above}\;\mathrm{canopy}}\times 100$$

At the anthesis stage in all seasons, we also measured the PAR intensity within the canopy at 0 (slightly above the soil surface), 15, 30, 45, and 60 cm above the soil surface. The relative PAR intensity at a specific canopy height was calculated as follows.

(7) $$\mathrm{Relative}\;\mathrm{PAR}\;\mathrm{intensity}\;\left(\%\right)=\frac{\mathrm{PAR}\;\mathrm{intensity}\;\mathrm{at}\;\mathrm{specific}\;\mathrm{canopy}\;\mathrm{height}}{\mathrm{PAR}\;\mathrm{intensity}\;\mathrm{above}\;\mathrm{canopy}}\times 100$$

The plant height was about 75 cm for wheat, so the PAR intensity at 75 cm above the soil surface was the PAR intensity above the canopy in practice.

The intercepted solar radiation (ISR) during a growth period was calculated using the average canopy PARI and accumulated incident solar radiation in the growth period, as follows.

(8) $$\mathrm{ISR}\left(\mathrm{MJ}m^{-2}\right)=\frac{\mathrm{PARI}\;\mathrm{at}\;\mathrm{the}\;\mathrm{beginning}+\mathrm{PARI}\;\mathrm{at}\;\mathrm{the}\;\mathrm{end}\;\mathrm{of}\;\mathrm{the}\;\mathrm{period}}{2}\times \mathrm{incident}\;\mathrm{solar}\;\mathrm{radiation}$$

The ISR during the entire growing season was the summed ISR during each growth period. The RUE during one period was calculated as follows.

(9) $$\mathrm{RUE}\left(g\;{\mathrm{MJ}}^{-1}\right)=\frac{\mathrm{Biomass}}{\mathrm{ISR}}$$

### 4.4. Data Analysis

Statistical analyses were performed with Statistix 9.0 (Analytical Software, Tallahassee, FL, USA). Analysis of variance was conducted separately in each year. Differences in yield and yield-related traits under NDS and WBS at the same seed rate were detected using the Student’s *t*-test (*α* = 0.05). Pearson correlation analysis was conducted to determine the correlation coefficient (*r*). All graphical representations of data were produced using SigmaPlot 12.5 (Systat Software Inc., Point Richmond, CA, USA).

## 5. Conclusions

Overall, WBS had a significant and positive effect on the grain yield (4.7–15.4%), but the underlying mechanism involved differed between seed rates. Yield increase by WBS was mainly attributed to the increase in total biomass resulting from both the promoted pre- and post-anthesis biomass production, except that only the increase in post-anthesis biomass mattered at the lowest seed rate (SR100). Before anthesis, the increased ISR led to the higher biomass. After anthesis, the increase in biomass was attributed to the increased ISR rather than RUE at low seed rates (100 and 200 m^−2^), for which the more homogeneous canopy was, whether mainly or partly, responsible. In contrast, the increased RUE instead of ISR worked at medium and high seed rates (400 to 700 m^−2^), where the enhanced PAR distribution within canopy and the increased ISR played an important role. The greatest increases in total biomass, pre-anthesis ISR, and post-anthesis RUE were all achieved at SR500, thereby obtaining the highest yield. Our research found that WBS enhanced grain yield by increasing ISR before anthesis and improving RUE after anthesis, and we highlighted that the relatively higher seed rates (400–500 m^−2^) should be adopted, to fully use the positive effect of WBS to maximize wheat yields.

## Figures and Tables

**Table 1:** Analysis of variance for yield and yield-related traits.

Season	Source	Yield	EN	GPE	GW	MSN	PSP	YPE	B_pre_	B_post_	B_total_	ISR_pre_	ISR_post_	ISR_total_	RUE_pre_	RUE_post_	RUE_seasonal_	HI	LAI_an_	SLN_an_
2020–	SM	**	**	ns	ns	**	ns	*	**	**	**	**	*	**	*	**	*	ns	**	ns
2021	SR	**	**	**	**	**	**	**	**	**	**	**	**	**	**	**	**	**	**	**
	SM × SR	**	**	ns	ns	**	ns	**	**	**	**	**	*	**	*	**	**	ns	**	ns
2021–	SM	**	**	ns	ns	**	ns	*	**	**	**	**	*	**	*	**	*	ns	**	ns
2022	SR	**	**	**	**	**	**	**	**	**	**	**	**	**	**	**	**	**	**	**
	SM × SR	**	**	ns	ns	**	ns	**	**	**	**	**	*	**	*	**	**	ns	**	ns

* and **, significant at 0.05 and 0.01 probability levels, respectively; ns, not significant at 0.05 probability level; SM, sowing method; SR, seed rate; EN, ears number; GPE, grains per ear; GW, grain weight; MSN, the maximum stem number; PSP, productive stem percentage; YPE, yield per ear; B_pre_, pre-anthesis biomass; B_post_, post anthesis biomass; B_total_, total biomass; HI, harvest index; ISR_pre_, pre-anthesis intercepted solar radiation; ISR_post_, post-anthesis intercepted solar radiation; ISR_total_, total intercepted solar radiation; RUE_pre_, pre-anthesis radiation use efficiency; RUE_post_, post-anthesis radiation use efficiency; RUE_seasonal_, seasonal radiation use efficiency; LAI_an_, leaf area index at anthesis; SLN_an_, specific leaf nitrogen content.

## Data Availability

Data are contained within the article.
